# Nitrogen Pollution Impact and Remediation through Low Cost Starch Based Biodegradable polymers

**DOI:** 10.1038/s41598-020-62793-3

**Published:** 2020-04-03

**Authors:** K. A. Ibrahim, M. Y. Naz, S. Shukrullah, S. A. Sulaiman, A. Ghaffar, N. M. AbdEl-Salam

**Affiliations:** 1 College of Engineering, Muzahimiyah Branch, King Saud University, Riyadh, 11451 Saudi Arabia; 2 Department of Chemical Engineering, Al-Hussein Bin Talal University, Ma’an, Jordan; 3 Department of Physics, University of Agriculture, 38040 Faisalabad, Pakistan; 4 Department of Mechanical Engineering, Universiti Teknologi PETRONAS, Seri Iskandar, 32610 Malaysia; 5 Arriyadh Community College, King Saud University, 11437 Arriyadh, Saudi Arabia

**Keywords:** Biophysics, Chemistry, Engineering, Materials science

## Abstract

The world does not have too much time to ensure that the fast-growing population has enough land, food, water and energy. The rising food demand has brought a positive surge in fertilizers’ demand and agriculture-based economy. The world is using 170 million tons of fertilizer every year for food, fuel, fiber, and feed. The nitrogenous fertilizers are being used to meet 48% of the total food demand of the world. High fertilizer inputs augment the reactive nitrogen levels in soil, air, and water. The unassimilated reactive nitrogen changes into a pollutant and harms the natural resources. The use of controlled-release fertilizers for slowing down the nutrients’ leaching has recently been practiced by farmers. However, to date, monitoring of the complete discharge time and discharge rate of controlled released fertilizers is not completely understood by the researchers. In this work, corn starch was thermally processed into a week gel-like coating material by reacting with urea and borate. The granular urea was coated with native and processed starch in a fluidized bed reactor having bottom-up fluid delivery system. The processed starch exhibited better thermal and mechanical stability as compared to the native starch. Unlike the pure starch, the storage modulus of the processed starch dominated the loss modulus. The release time of urea, coated with processed starch, remained remarkably larger than the uncoated urea.

## Introduction

### Nitrogen cycle and environmental pollution

The world is facing the problem of exponential population growth. The population of this planet was around 3 billion in 1960, which is expected to reach 9 billion in 2040^1^. The world does not have too much time to ensure that there is enough land, food, water, and energy for the fast-growing population. The United Nations warns that if humans remain unsuccessful in curbing overpopulation, more than three billion people will be in poverty in coming years. The growing food crisis also presents an opportunity for researchers and investors in the farming sector, especially in fertilizer production, to invest more in this profitable business. The rising food demand has also brought a positive surge in the fertilizer demand. Due to large input of mineral fertilizers, the global production of crop and livestock has increased significantly over the past century. As revealed by the International Fertilizer Association, the world uses 170 million tons of fertilizer every year for food, fuel, fiber, and feed. Among this, the nitrogenous fertilizers are being used to meet 48% of the total food demand of the world^2^. Over-fertilization augments the pollutants’ level in the soil, air and water. The excessively available nitrogen during fertilization of crops also contributes to environmental pollution. The unassimilated reactive nitrogen acts as a pollutant and harms the natural resources.

**Figure 1:**
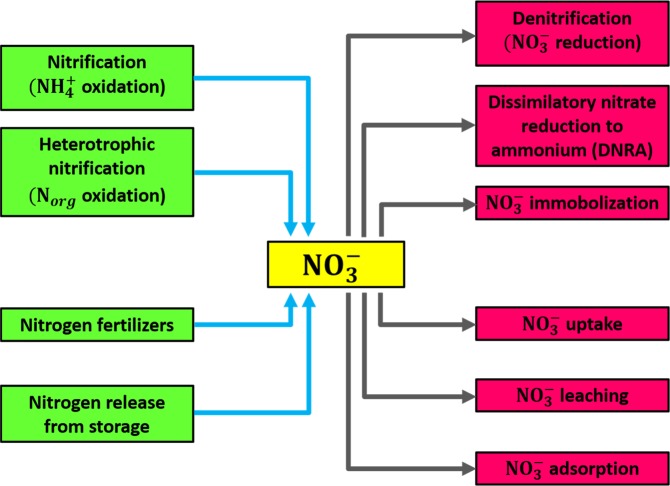
Nitrogen cycle in terms of major biological pathways for ${\boldsymbol{N}}{{\boldsymbol{O}}}_{3}^{-}$ reduction.

Most of the pollution in the world today is caused by human beings. The contribution of nitrogen to the environment pollution is increasing with growing human population. Sources of manmade pollution include excessive fertilization of crops, sewage, stormwater, industry, automobiles, burning of wood and fuels, etc^3^. The nitrogen cycle in terms of fixation, ammonification, nitrification and denitrification is explained in Fig. 1. The major part of the nitrogen primarily comes from the industrial nitrogen fixation, which directly contributes to the nitrogen in terrestrial ecosystems. This increase in nitrogen in the environment is a source of high deposition of nitrogen in agriculture-dominated landscapes^1,4^. Nitrogen fertilizers, nitrogen in foods and airborne nitrogen emissions are the three main sources of reactive nitrogen. The food products represent the major part of reactive nitrogen (38–75%) in the world. The airborne emissions of ammonia and nitrogen oxides, and the subsequent deposition from the atmosphere contribute about 11–36%, while nitrogen fertilizers contribute about 11–32% to the reactive nitrogen^5^. The surplus reactive nitrogen can cause pollution problems in the environment, such poor air quality, acidification of lakes and rivers, disruption of foresting process and degradation of coastal water quality.

A core process in nitrogen cycle is denitrification, a heterotrophic process carried out by facultative anaerobic organisms which use various C substrates as donors of electrons^6,7^. A better understanding of denitrification and dynamics of nitrogen transformation associated with it are crucial. Nitrate is the main nitrogen species for loss of nitrogen and is influenced by several nitrogen flows that produce and consume $N{O}_{3}^{-}$ via nitrification and leaching, respectively. To be environmentally friendly, $N{O}_{3}^{-}$ must be reduced to a nonreactive form (N_2_). The key biological pathways of $N{O}_{3}^{-}$ reduction, as shown in Fig. 1, are (i) assimilatory $N{O}_{3}^{-}$ reduction into biomass, (ii) dissimilatory $N{O}_{3}^{-}$ reduction to N_2_ and (iii) dissimilatory $N{O}_{3}^{-}$ reduction to $N{H}_{4}^{+}$^8^. Dissimilatory reduction can result in denitrification under limited conditions and provide an energetically favorable alternative to denitrification^9^. Nevertheless, there is still a need for further work to recognize the importance of DNRA in terrestrial systems and to understand perfect methods for studying the mechanism.

Researchers are trying to investigate the different methods and mechanisms to minimize nitrogen losses, particularly due to fertilization. The performance of a fertilizer can be enhanced through many ways, including nitrification inhabitation, urease inhabitation and controlled-release fertilizer. Farmers around the world have recently explored the use of controlled-release fertilizers to slow down leaching of the nutrients. The added advantage of such fertilizers is the controlled availability of nutrients in the soil for longer time periods. The acceptable properties of a fertilizer to qualify as a controlled-release fertilizer vary from researcher to researcher. Under ambient conditions and absence of any external stress, Trenkel guidelines for slow release fertilizer are: (i) no more than 15 percent release within 24 hours, (ii) no more than 75 percent release within 28 days, and (iii) at least about 75 percent release after specified timeframe^10^. Ţolescu and Iovu^11^, revealed that a controlled-release fertilizer contains at least one nutrient, which delays its availability to plant after application, or available to plant over a substantially longer period of time than a normal quick release fertilizer. Shaviv^12^ criteria for a controlled-release fertilizer is that the factors affecting the release rate, release pattern and release time should be well understood and controllable during preparation of a fertilizer.

### Nitrogen pollution control by coating urea

A controlled-release fertilizer is generally produced by coating the granular fertilizer and producing a physical barrier at the surface to control the water penetration into the core. The release of nitrogen from the coated core slows down and the farmer gets good fertilizer performance. One determined advantage of the coated fertilizers is availability of the nutrients to the plant for longer periods of time. The rate of nutrients’ leaching normally depends on properties of the material. The common materials, reported in the past literature, are neem, resins, sulfur, natural carbohydrate polymers and synthetic polymers^13,14^. The coated layer acts as a semipermeable or impermeable membrane having tiny pores. This membrane temporarily isolates the core from the surrounding environment^15^. The leaching of nutrients from the membrane barrier depends on the properties of the material^16,17^. The leaching process is not much affected by salinity, pH, texture, microbial activity and cation exchange capacity of the soil. It reveals that monitoring of nutrients’ leaching from the coating membrane is not a trivial case.

Many research efforts are underway to understand the release time, release rate and mechanism of interaction of coating with water in the soil. Sulphur coated fertilizers are costly and the coating cracks easily because of its friability^18,19^. Starch, lignin and cellulose have limited controlled release characteristics due hydrophilicity^20–22^. Other coated fertilizers use thermoplastics, ethylene-vinyl acetate or surfactants as diffusion retardent materials. Some zeolite-based controlled-release fertilizers have also been developed. Bansiwal *et al*.^23^ used surfactant-modified zeolite to coat Phosphorus fertilizers. Notario *et al*.^24^ prepared Phosphorous and Potassium slow release fertilizers from concentrated solutions of Potassium Dihydrogen Phosphate and Dipotassium Phosphate. Other coating materials include soda flax lignin^25^, graphene oxide films^26^ and gypsum plaster^19^. Gonzales *et al*.^27^ reported some polymeric materials for slowing down the urea hydrolysis. These polymers include ethyl cellulose, acetate cellulose and sodium alginate. Solihin *et al*.^28^ incorporated K^+^, NH_4_^+^ and PO_4_− ions in kaolin structure as nutrients for plant. Costa *et al*.^29^ coated urea with polyhydroxybutyrate and ethyl-cellulose in the presence of different emulsifiers.

Polyurethane is also a versatile polymeric material first developed in 1930s for use in aerospace and military industries^30^. The high toughness, chemical resistance, flexibility and abrasion resistance of polyurethane make it an attractive material for coating applications. The reported literature shows that materials containing urea or urethane groups within the backbone are gaining more significance in coating industry^31^. Although, synthetic polymeric coating materials have shown several merits over natural polymers^32^, these materials form impermeable or semi-impermeable membranes of tiny pores. The main issue in producing the polymer coated urea is the choice of right material for coating and the associated coating process^33^. The release of nitrogen through a membrane is mainly influenced by physical and moisture permeability properties of the coating. The soil properties did not influence much the release rate. The moisture permeability of the coating materials may be regulated by altering their composition. Therefore, the nutrient release from a polymer-coated urea can be predicted much more accurately over a given period of time than that covered with inorganic materials^12,33^. Most of the used polymers, however, are quite costly and non-degradable, so emphasis should be on low-cost, environmentally friendly polymers. The presented work is focused on the thermal processing of corn starch into a coating material using di-sodium tetraborate and urea.

## Materials and Methods

### Preparation of coating solution

Corn starch is a low cost, biodegradable and eco-friendly carbohydrate polymer. In the presented work, processed corn starch was used to produce controlled-release urea. To reduce the process cost, the starch with terminated shelf life can also be tested for the stated purpose. Since pure starches show poor mechanical strength, tacking ability and viscosity, they can not be used to produce good water retardant coatings^34^. The specific rheological properties, needed to improve the adhesion and coating properties, are often incorporated through chemical modification of the starches. Chemical modification of the starch produces esters and ethers^35^. A chemical reaction also gives anionic or cationic character to the starches. Through chemical modification, the starches receive additional properties enabling their applications in foods industry and various technical sectors.

Comprehensive alteration of physical and chemical properties of pure starches is possible through their reaction with various chemical regents. These chemical additives convert a pure starch system into a long chain biopolymer complex of improved viscosity, tacking ability, mechanical strength and surface tension. The interactions can be intra or intermolecular or both, depending on nature, length, groups, hydration capacity, degree of polymerization, salinity, pH and co-solvents. Since polymeric chains in a starch are more flexible than polymeric chains in cellulose, starches are highly soluble in many solvents^25,36^. In the given work, de-ionized water was used as a standard solvent. The solute was a premix of corn starch, urea and borate (Na_2_B_4_O_7_.10H_2_O). Corn starch was commercial grade product whereas the Analytical Reagent (AR) grade chemicals were supplied by R and M Chemicals. Four compositions of the coating material were formed and tested for their rheological, physical and slow release coating properties. The coating material was prepared by adopting the procedure of Naz *et al*.^37^. The details of formulation of coating material and processing temperature are presented in Table 1.

**Table 1:** Composition of the coating material.

Solution	Water (ml)	Starch (g)	Urea (g)	Borate (g)	Reaction Temp. (°C)
S_0_	1000	50	0	0	80
S_1_	1000	50	15	0	80
S_2_	1000	50	0	4.5	80
S_3_	1000	50	15	4.5	80

### Coating experiments

**Figure 2:**
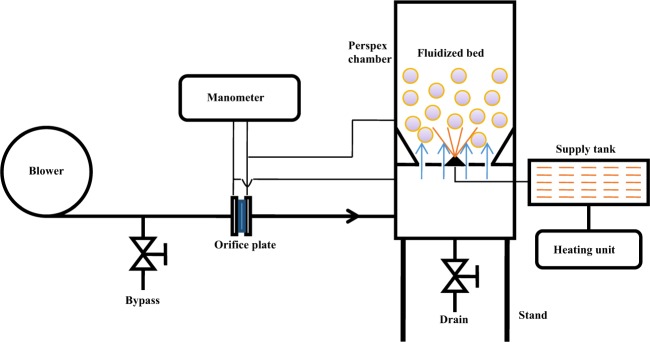
Schematic illustration of fluidized bed spray coating process.

Once the physical parameters of the coating material were fully depicted, it was coated over granular urea in a conventional fluidized bed spray coater. In the coating reactor, as shown schematically in Fig. 2, the atomizer was mounted at the base of the fluidized bed column. A small distance of 80 mm was maintained between atomizer and granular urea in its fluidized state. This approach minimizes drying of the spray and increases uniformity of the coating. Since the pneumatic mass flow provided the high kinetic energy from the bottom of the bed, the wetted particles tended to fluidize to produce evenly coated urea. An air blower was used to fluidize 500 g of urea above the minimum level of fluidization. The prepared coating material was sprayed from the bottom to coat the fluidized urea particles. A full cone spray nozzle was used to spray the coating material at a temperature of 80 °C and pressure of 5 bar. The coated urea was dried at 60 °C at the end of the coating process to remove the moister.

Both the uncoated and coated urea samples were inspected for coating thickness, percentage of coating material, coating morphology, nutrient discharge rate, complete dissolution time, and coating strength. Scanning electron microscopy was used to elaborate the surface morphology and coating thickness of the coated urea. The crushing strength of the urea samples was measured with a tablet tester. The crushing strength was measured in terms of constant force (N). The force required to crack the coating is called coating sensitivity. A dissolution rate test was conducted to measure the discharge time of the uncoated and coated urea. In this test, the urea was released in distilled water under a shear rate of 200 rpm. Both coated urea and control were weighed to 10 g and placed in separate glass beakers. A total of 200 mL of deionized water was added to the sample and the urea-water mixture was stirred at room temperature by using an overhead stirrer. The time for complete release of urea in distilled water was noted.

### Ethical approval

This article does not contain any studies with human participants or animals performed by any of the authors.

## Results and Discussion

### Rheological traits of coating solution

**Figure 3:**
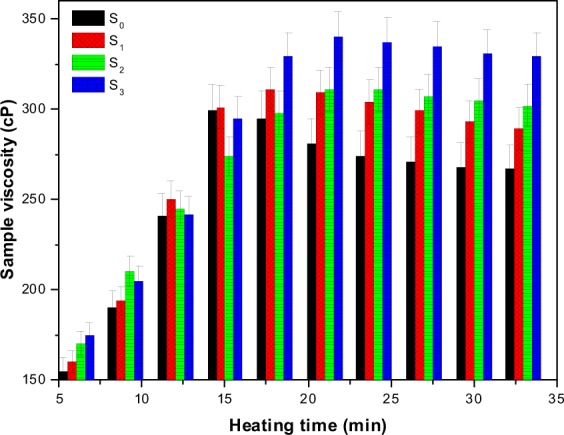
Viscosity profiles of pure and modified starch.

The processing conditions notably change the time and temperature of gelatinization of the starch suspension^38^. The possible changes in the viscosity of the modified and native starches over time are reported in Fig. 3. After 15 min of heating, S_0_ sample achieved maximum viscosity at a constant heating temperature of 80 °C. The viscosity of the native starch suspension was reduced by 34 points when the suspension was further heated after 15 min. After 25 min, the suspension viscosity remained unchanged over time. the peak viscosity of S_0_ was measured about 300 cP. Other samples also showed similar viscosity plots. The peak viscosity of S_1_, S_2_, and S_3_ samples was found relatively higher than the pure starch. The viscosity curves of these samples attained the peak values slightly later than the peak value of native starch. The lessening of viscosity of the modified starch after attaining the peak value had not been as noticeable as in case of pure starch.

**Figure 4:**
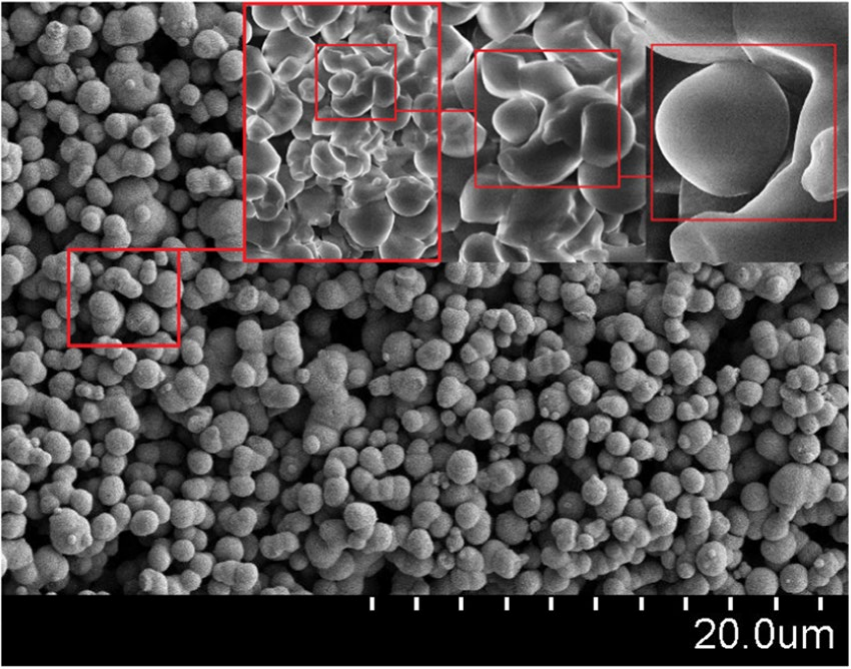
SEM illustration of deformation and swelling of corn starch.

**Figure 5:**
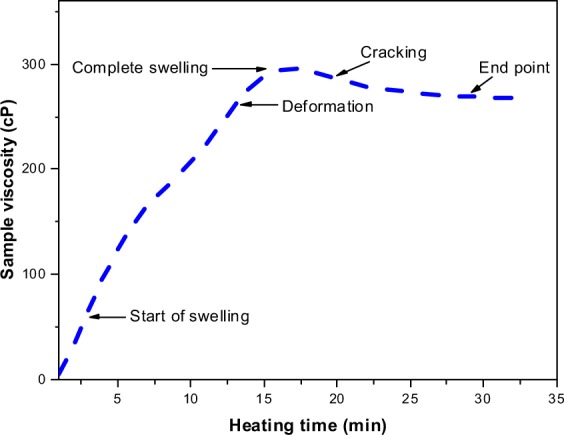
Illustration of stages of corn starch processing.

The alleviation of viscosity with time from the peak value might be ascribed to high process temperature and prolonged heating of the suspension. Because the temperature of decomposition of the starch granules is reported as lower than its melting point, the granules rapidly expand and crack over time. Figure 4 depicts the swelling of the starch granules in the heated suspension. Upon cracking, the suspension loses its tightness, and consequently the viscosity. The deformation and cracking of the starch particles over time is illustrated in Fig. 5. The viscosity profile reflected the possible variations in the granule’s shape during the processing of the starch. The viscosity profile exhibited a linearly increasing trend at early stages of starch processing due to swelling of the granules and amylose leaching. After 7 min of heating, the starch granules completely swelled out and the viscosity profile reached the peak point. Beyond this point, heat treatment of the suspension resulted in a viscosity breakdown due to the shear field of the instrument and consequently a decrease in viscosity^39^.

The suggested viscosity breakdown in Fig. 5 can be avoided by introducing cross-linking and plasticizing agents in the suspension^40^. Figure 3 suggests that the presence of borate and urea in the formulation caused a small change between the peak viscosity and the final viscosity of the chemically altered starch. These modifiers notably improve the stability of the suspension by reducing the starch cracking. In response, the suspension retains its tightness and therefore viscosity breakdown was not as detrimental as it was in pure starch. The S_3_ sample gained the highest viscosity among the investigated samples. The high viscosity was attributed to addition of di-sodium tetraborate in the dispersion, which dissociated into borate and sodium ions. The reactive borate ions formulated the polymer chains through hydrogen bonding with the starch whereas the charge on the developed chains was shielded by the free Na^+^ ions.

**Figure 6:**
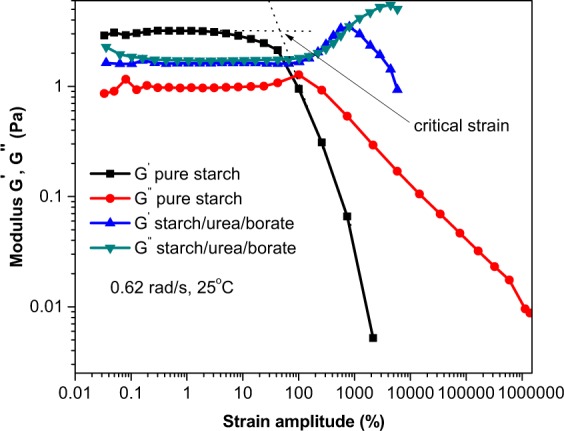
Strain sweep for determination of critical strain at room temperature.

**Figure 7:**
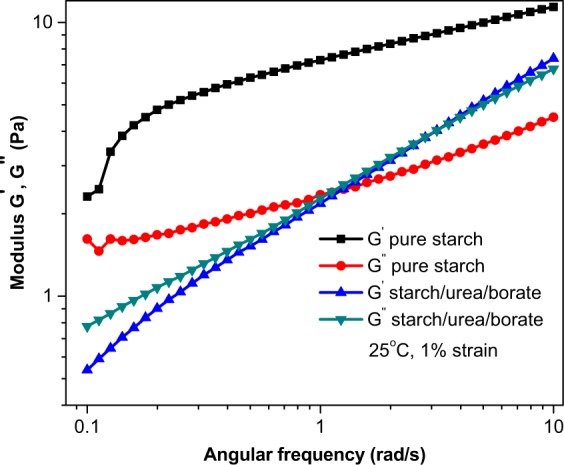
Frequency sweep: storage and loss moduli as a function of angular frequency.

A comparison of the storage modulus of S_0_ and S_3_ samples is provided in Fig. 6. The time-based storage modulus plots revealed high stability of S_3_ over S_0_. The pure starch destabilizes after 300 seconds, which suggests low stability of material over time. The gel of native starch breakdowns more sharply over time. In contrast, the modified samples retained their gel structures over longer periods of time. Strain sweep also reveals that after critical strain, the samples behave like a fluid (G′ < G″). It is an indication that beyond the critical strain, the material response is more complex and is no longer a function of strain alone but G′ and G″. Figure 7 provides a comparison of frequency sweep responses of native and modified starch at 1% strain. The frequency sweep profiles provide information about the storage and loss moduli with a change in angular frequency. The storage modulus of the modified starch was reported higher than the loss modulus, which confirms the gel formation character of the modified starch and dense fluid-like character of the pure starch. It was worth noting that the magnitude of G′ and G″ for strong gel should reach up to one million. Because the magnitude of G′ and G″ of the modified starch was not as high as required for a strong gel, it was regarded as a weak gel. The unmodified starch sample was fluid in nature where the G″ component dominated the G′ at lower angular frequencies.

### Surface morphology

**Figure 8:**
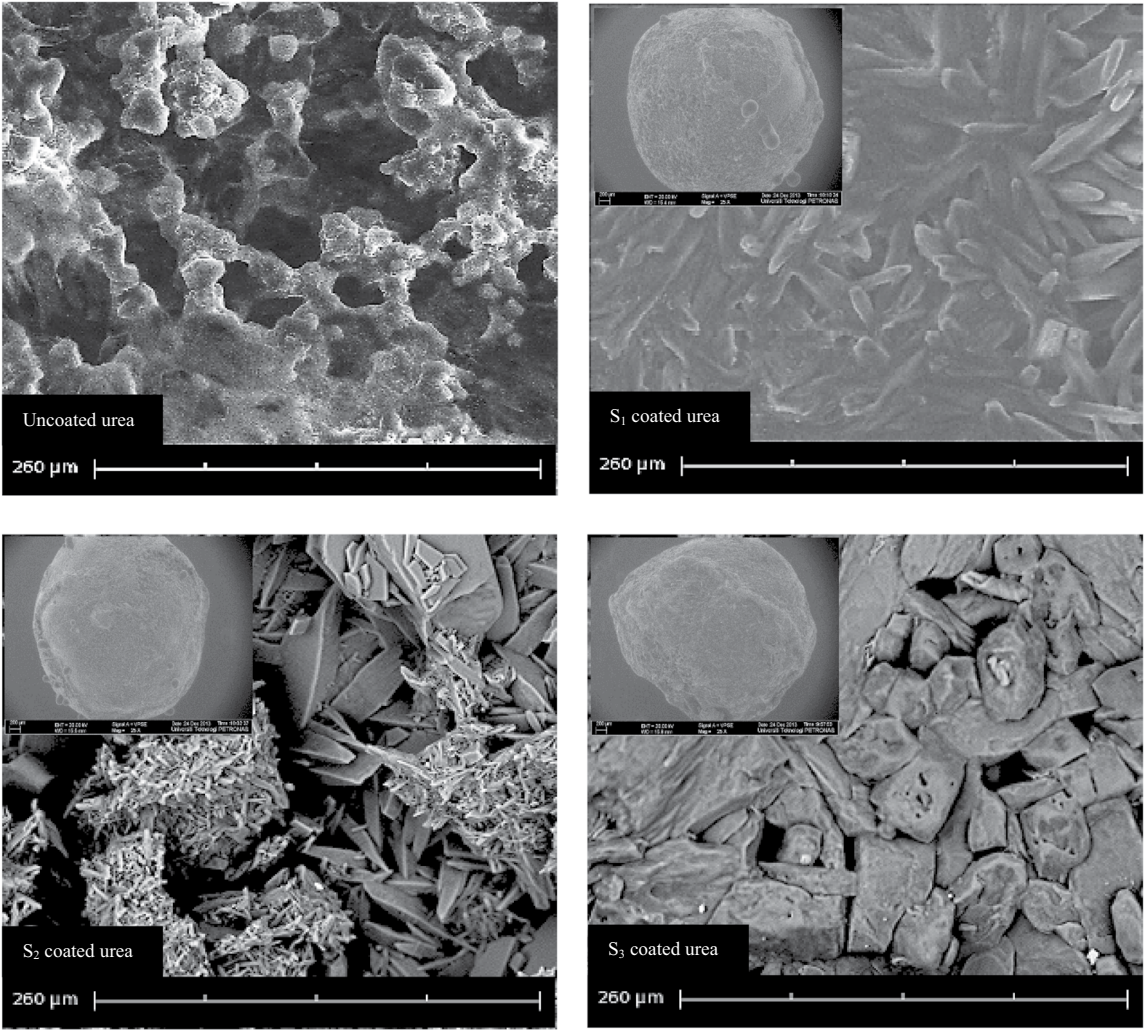
SEM micrographs of uncoated urea and coated with processed corn starch.

The coating content typically accounts for 3 to 15% of the weight of the finished product^10^. The amount of coated material influences the surface morphology and release parameter of the product. The coating content depends on the physical and chemical traits of the coating material and the process parameters. In this work, the coating morphology and surface properties of the finished product were studied from SEM micrographs. As a thick layer of coating material was formed on the surface of urea granules, 20 granules from each sample were randomly selected and scanned through SEM technique. Figure 8 shows SEM micrographs of surface of coated and uncoated urea.

High surface roughness was seen in SEM micrograph of uncoated urea. Without coating a layer, the surface was less rigid and highly porous. Compared to uncoated urea, surface of the coated urea was highly dens and smooth. The coated surface also looked hard, uniform, compact and water resistant. SEM micrographs of the samples, coated with S_0_, S_1_, S_2_ and S_3_ materials, clearly differ in terms of surface morphology. As shown in Table 2, the mean thickness of the coating was determined by taking the difference in diameters of uncoated and coated granules. Each measurement was carried out by taking 10 coated granules of roughly same size from each sample and noting their diameters from the respective SEM micrographs. In addition, the coating thickness was also evaluated manually using a Vernier Caliper. The S_0_ material produced the thin coating layer, whereas S_3_ produced the thickest coating layer among the tested materials. The coating percentage of S_3_ material was higher than all other tested materials.

**Table 2:** Effect of composition on coating thickness and percent coating.

Solution	% Coating	Thickness (mm)
S_0_	2.84 ± 0.240	0.21 ± 0.010
S_1_	3.67 ± 0.201	0.28 ± 0.012
S_2_	3.73 ± 0.107	0.30 ± 0.015
S_3_	4.32 ± 0.243	0.47 ± 0.017

### Coating strength release test

**Figure 9:**
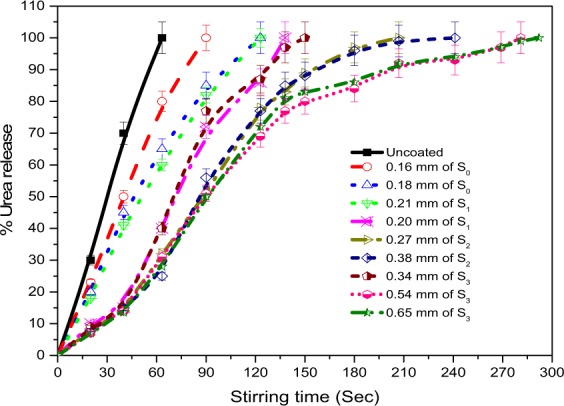
Release time of coated and uncoated urea.

Different methods are available for testing the controlled release fertilizers, including the laboratory, greenhouse, growth chamber and field methods^41,42^. The researchers are still designing a standardized method for commercial purposes to test the slow release fertilizers. The laboratory methods under controlled conditions are however the best and easiest way of screening the slow release fertilizers quickly^11^. In this work, dissolution rate testing of the coated and uncoated urea was performed in water. In a beaker, 10 g of each urea sample was taken, and 200 ml of deionized water was also added to the system. At room temperature, an overhead stirring at a constant speed of 200 rpm agitated the water-urea mixture. The time of complete dissolution of each sample was noted, as shown in Fig. 9. Compared with coated ones, nitrogen began to discharge immediately from the uncoated urea. The maximum time of complete dissolution of uncoated urea was 65 seconds. The release time was considerably increased with coating thickness in the presence of chemical additives. A 0.54 mm thick coating gives optimum release time. The overall discharge duration of urea coated with S_3_ material was relatively greater than those coated with other rest of the materials. Shi *et al*.^43^ coated the urea with a mixture of plastic and starch and revealed prolonged nitrogen release time as compared to the pure starch coating.

In addition to the above stated parameters, the coated product should also show adequate mechanical resistance to normal handling and storage for avoiding the surface fractures^44^. The mechanical resistance was measured by firmly pressing on the individual coated granules and observing the surface cracks. The mechanical strength greatly depended on the coating composition and thickness. The uncoated urea granules and those coated with S_1_ and S_2_ exhibited very low mechanical strength as compared S_3_ coated urea. In addition, the surface strength was considerably improved with an increase in the coating thickness to a certain point (0.54 mm), after which the coating thickness was not significantly influenced. The overall mechanical strength of coated urea varied from 22 N to 20 N. The uncoated urea exhibited the coating strength of 20 N.

## Conclusions

High urea inputs raise the level of reactive nitrogen in the soil, air, and water. Unused reactive nitrogen acts as a pollutant and harms the natural resources. The use of controlled-release fertilizers for slowing down the nutrients’ leaching has recently been practiced by farmers worldwide. The added advantage of such fertilizers is the availability of controlled amount of nutrients in the soil for longer periods of time. However, to date, monitoring of the complete discharge time and discharge rate of controlled released fertilizers is not completely understood by the researchers. The starches, modified with urea and borate, showed good stability and mechanical strength over time. A decrease in the storage modulus of the native starch showed unstable gel structure, which may break after some time. The viscous component dominated the elastic component at lower angular frequencies for the native starch. A small difference between the peak and end point viscosities of the modified starch suggested that the presence of urea and borate in the starch suspension considerably reduced the starch cracking. The S_3_ coated urea gained the highest viscosity among the investigated samples. SEM analyses showed a less dense surface morphology of the uncoated urea with a high degree of roughness. The porosity of the uncoated granules was also quite high compared to the coated granules. The surface of the coated granules was uniform, dense, and hard with low porosity. Fast release of the uncoated urea was predicted compared to the coated samples. The uncoated urea was completely released into water after 6 min. Conversely, the slowest release was predicted from the urea coated with the S_3_ material. The coated granules exhibited the highest crushing strength of 30 N, which was noticeably higher than the crushing strength of the uncoated urea (20 N).
